# Grand Challenge: Computational Models Validated Against Critical Experiments

**DOI:** 10.3389/fbioe.2013.00001

**Published:** 2013-06-12

**Authors:** Jack C. Roberts

**Affiliations:** ^1^Applied Physics Laboratory, Johns Hopkins University, Laurel, MD, USA

There are many areas in biomechanics that could be considered a “Grand Challenge,” I have selected, as a Grand Challenge, the development of computational models of biomechanical systems from the sub-cellular level to the whole body that have been properly validated against critical experiments. The types of models can be best shown in a table that lists the anatomies and the processes that are involved, see Table 1.

**Table 1:** **Anatomy and processes for computational modeling**.

Anatomy	Process				
	Transport	Force/stress	Deflection	Injury	Modeling approach
Whole body	Cardiac output, lung physiology,	Force/pressure impacts, acceleration	Dislocation, falls, blunt trauma, whole body vibration	Movement or behavior disorders	Lumping of different models
Region	Blood/lymph flow (intracranial, cardiac, pulmonary). Lung collapse	Forces/pressure, impacts, acceleration, force transmission	Blunt trauma, penetrating trauma, impact, whiplash	Reflex sympathetic dystrophy, compartment syndromes, lower extremity injuries	Lumping of different models
Organ	Circulation, interstitial pressure, intracranial pressure	Energy/forces, pressures applied to organs	Relative motion between organs and bones, peak deflections in bones	Ruptured spleen, lung, or brain contusions. TBI,	Lumping of different models
Tissue	Regulation of circulation, pO2, pH, edema	Stress, strain energy density	Strain peaks	Extracellular damage, fracture, edema	Continuum
Cells	O_2_, nutrients	Membrane tension, filament forces, dermatomes	Cell stretch, axonal stretch, smooth muscle contraction	Rupture, apoptosis	Sub-continuum
Subcellular	ATP	Stress	Actin/myosin/tubulin	Membrane stretch	Lumped sub-continuum

Most successful modeling efforts are focused on answering well-defined questions, and as such they are focused on specific physiologic processes that are considered within a hierarchy of anatomic detail. For example, an impact to the head can be considered from a system level, considering head accelerations and neck constraints, and resulting in an index such as a Head Injury Criterion (HIC) score. A head impact can also be considered from an organ point of view, addressing bone flexure, considering anatomic variations, and resulting in a likelihood of facial or cranial fractures. Further, head impacts could be considered from a tissue level, addressing strain distributions and peaks, and considering anatomic features such as white/gray matter, sulci, and internal membranes in the brain. Head impacts can also be considered from an injury process point of view, addressing the physiologic response to mechanical damage, and the likelihood of minor bleeding, edema, or circulatory changes. Finally, head impacts could be considered form a cellular or sub-cellular point of view, assessing axonal patency, membrane rupture, or apoptosis. In fact, head impact as a process crosses all these modeling hierarchies, and any modeling effort should address these hierarchies of anatomy, either justifying the relevance of the model or clarifying the assumptions that make the modeling effort meaningful to answer the questions posed. Clearly, an integrated understanding of a process such as head injury involves an iterative effort whereby the assumptions for the modeling efforts at each hierarchical level are revisited, and the relevance of various features are re-assessed as overall understanding improves. Therefore, Table 1 shows a conceptual relationship between anatomic hierarchies and some generic physiologic processes, with examples of the processes that occur at the different anatomical levels. A generic force-dominated process is shown, and a generic displacement-dominated process is also shown, along with examples of the types injuries that focus attention on the hierarchies considered.

With the appropriate caveats, research at each level can be well-justified. For example, a tissue-level (strain-based) study of brain injury could assume that any tissue damage at the cellular level does not affect the continuum-level stress-strain relationships used; it would assume that the structure of the head is relevant (i.e., that the head considered is either an accurate computational model, or that the animal model used is relevant); and it would assume that the overall forces applied to the head are either accurate for humans or scaled appropriately.

A number of such models at different levels have already been developed and validated under differing conditions, but there are no full body human models that include full validation at all levels shown in Table 1. Probably the most complete full body human finite element (FE) model is that developed as part of the Global Human Body Models Consortium (GHBMC) by Gayzik et al. (2012). This model includes the skeletal structure and all organs and has been validated against a number of frontal and lateral rigid impactor and sled tests. This model does not include anatomic detail below the organ level in Table 1. A total human model for safety (THUMS) FE model was developed, primarily to study various kinematic injury mechanisms, and is used as a substitute for the crash test dummies used for car occupants and pedestrians, Iwamoto et al. (2002). This model was validated through the verification of pedestrian's whole body kinematics and lower extremity injuries, but lacks the detailed definition of material properties and anatomic detail in order to predict injuries in the organs.

Human torso models, as an examples of a regional model, with varying degrees of anatomic detail, have been developed and validated under differing conditions, from blunt to ballistic impact and blast, see for instance, Chen (1978), Plank and Eppinger (1989, 1991), Plank et al. (1994), Wang (1995), Jolly and Young (2000), Shen et al. (2008), and Roberts et al. (2007). There have also been human head FE models, of varying anatomic detail, developed and validated to compute strains and intracranial pressures in order to assess whether there have been focal injuries (contusion) or Diffuse Axonal Injuries (DAI). These models have been validated with forcing functions that include head linear and rotational acceleration as well as blunt impact and blast, see for instance, Merrill et al. (1984), Dimasi et al. (1991), Trosseille et al. (1992), Ruan et al. (1993), Kang et al. (1997), Claessens et al. (1997), Kleiven (2002), Willinger and Baumgartner (2003), Takhounts et al. (2003), Deck et al. (2004), and Roberts et al. (2012).

Since “blast lung” has been thought to be one of the primary injuries to humans in blast events, there are organ level models that examine the effects of blast on the pulmonary system, see for instance, Stuhmiller et al. (1996). Other organ level models that have not been developed for blast and are either deterministic or hierarchical parametric probabilistic models and are, for instance, those of the: pulmonary system, Gayzik et al. (2011); aorta/heart, Shah et al. (2001); abdomen, Lee and Yang (2001); shoulder, Iwamoto et al. (2000); cervical spine, Nicolella et al. (2006); lumbar spine, Guan et al. (2006); lung, Vawter (1980), and those of the ribs, Li et al. (2010).

Tissue-level models that have been developed include, for instance: those on the heart, Shim et al. (2012) and Kerckhoffs (2012) and those of the brain, Prange and Marguilies (2002), Brands et al. (2002), and Arbogast et al. (1995). There are also cellular and sub-cellular level models of, for instance, alveolar cells and actin networks, Dailey et al. (2009); actin filament networks, Plamer and Boyce (2008), Gardel et al. (2004), and Unterberger et al. (2012); worm-like chains in a 3-D framework, Ogden et al. (2006), and multi-scale continuum modeling from the molecular to sub-cellular level, Cheng et al. (2012).

In broad terms, the models reviewed above advance understanding by either proposing mechanisms that explain the behavior of the processes that are studied, by showing that a particular set of mechanisms does not explain the process being considered, or by uncovering gaps in knowledge that are necessary to understand a process. However, many of the models in the literature suffer from two major weaknesses; they are either overly broad, and they produce predictions so generic that they cannot be tested in any meaningful sense (i.e., they have enough adjustable parameters to “fit” any behavior at all) or they are not tested against realistic data, so they do not reach a meaningful answer to the question of whether they represent the physical process they are aimed at. This makes the process of integrating models for an overall understanding of the physiology behind a large-scale process very difficult. Also, if a change in properties at the cellular level does affect the behavior at the continuum level, then multi-scale models that can span levels from the continuum to the nano-level need to be developed. These models would incorporate the individual fibers in a collagen matrix represented at one scale, and then all of the fiber homogenized to produce a bulk-level material property at a larger scale. When the model changes at the bulk level, the individual fibers are affected, changing (perhaps) the anisotropic behavior.

Therefore, a number of models now exists at each of the different levels shown in Table 1, but they are not validated for all of the loading scenarios and no effort has been made, at this time to link the different types of models, i.e., computational fluid dynamics (CFD) models, FE models, etc. This is a major task and could certainly be interpreted as a Grand Challenge because of all the different types of models, with differing degrees of validation for each. The grand challenge that this journal seeks to address is to provide a forum for models that are tested against experimental data in critical ways; whether they succeed in representing the experimental data or not, the comparison between proposed mechanisms and data will advance our understanding.
